# A Physical Model to Describe the Motion Behavior of BNNSs under Nanosecond Pulses

**DOI:** 10.3390/nano13071278

**Published:** 2023-04-04

**Authors:** Liang Zhao, Lin Zhou, Lin Yi Jin

**Affiliations:** 1 Key Laboratory of Advanced Science and Technology on High Power Microwave, Northwest Institution of Nuclear Technology, Xi’an 710024, China; 2 Institute of Nuclear Physics and Chemistry, China Academy of Engineering Physics, Mianyang 621999, China; 3 School of Design and Creative Arts, Loughborough University, Loughborough LE11 3TU, UK; L.jin@lboro.ac.uk

**Keywords:** BNNSs, global alignment, nanosecond pulses, kinetic model

## Abstract

This paper presents a physical model that provides a comprehensive understanding of the motion behavior of boron nitride nanosheets (BNNSs) immersed in ultrapure deionized water and subjected to a series of nanosecond pulses. In a study conducted by Y. Mi et al. The authors explored the global alignment behavior of BNNSs and fitted the experimental data with an exponential decay function. However, this function lacks clear physical mechanisms and the significance of the fitting parameters remains unclear. To address this issue, we have developed a kinetic model that explicitly describes the underlying physical mechanisms. Furthermore, we propose a simplified mathematical model that not only predicts the displacement of BNNSs but also estimates the total time, velocity, and acceleration of the motion process.

## 1. Introduction

**Figure 1:**
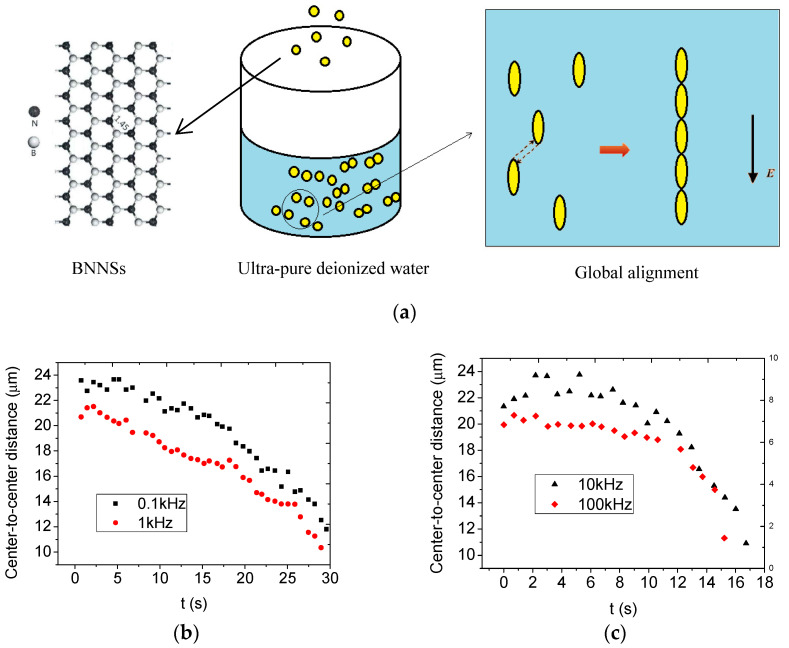
Schematics of global alignment of BNNSs under nanosecond pulses. (**a**) is the experimental setup and global alignment phenomenon. (**b**,**c**) are the experimental results under 0.1, 1, 10 and 100 kHz [4], respectively.

Polymer-based nanocomposites exhibit remarkable insulation properties, mechanical strength, and thermal conductivity, rendering them a highly promising avenue for research in the materials science domain. Consequently, research in this area has garnered considerable attention and remains a prominent area of focus [1,2,3,4,5,6,7,8,9]. Boron nitride nanosheets (BNNSs) are the two-dimensional counterpart of spherical graphene, with a B–N bond length of 1.45 Å, whereas graphene consists of carbon atoms arranged in a hexagonal lattice (1$\overset{o}{A}$ = 10^−10^ m), as seen Figure 1a. Therefore, BNNSs exemplify numerous distinctive properties [10], including elevated temperature stability, exceptional mechanical robustness [11], expansive band gap, antioxidation ability, and corrosion resistance. These singular attributes underscore BNNSs’ potential for several promising applications, spanning food processing [12], sensor manufacture [13], and drug and medicine fabrication [14], among others. Current research on BNNSs encompasses the synthesis [15], modification [16], and exfoliation of these materials [17].

Exposing BNNSs to nanosecond electric pulses has recently been demonstrated as an effective method for investigating their fundamental properties [4,5,18,19]. The use of nanosecond pulses minimizes the heat accumulation effect, enabling researchers to focus on the electrical response of BNNSs. Furthermore, the discrete nature of nanosecond pulses results in only small displacements of BNNSs, facilitating successive recording of their global movements.

Y. Mi et al. [4,5,18,19] have conducted numerous studies of this nature, wherein they have discovered an intriguing occurrence pertaining to BNNSs. Specifically, under nanosecond pulses, a singular BNNS will rotate locally to align its long axis with the applied field. Subsequently, multiple BNNSs will connect head-to-tail, ultimately forming a straight line in tandem with the direction of the applied field globally [4,5], as depicted in Figure 1a.

They used the center-to-center distance (*d*) of two BNNSs to depict this global alignment and fitted the experimental data of *d* on time (*t*) with an exponential decay function as follows [4]:

(1) $$d=d_{0}+Ae^{-\frac{t}{n}}$$

where *d*_0_, *A*, and *n* were fitting parameters. The fitting curve agrees well with the experimental data, as shown in Figure 1b,c. However, the potential physical mechanism was not well-disclosed by this equation. In addition, the physical meanings of the fitting parameters such as *d*_0_, *A*, and *n* were not clear at all.

In view of this, the motion process of BNNSs under nanosecond pulses is reanalyzed. A kinetic model to describe the relation between *d* and *t* is constructed, which not only gives good fitting, but also presents a clear physical picture of the global alignment behavior of BNNSs under nanosecond pulses. Aside from this section, Section 2 is devoted to the strict physical model. Section 3 is devoted to a simplified mathematical model. Section 4 is for the application of this simplified model. Section 5 deals with remarks on this model. The last section is for the conclusion of this paper.

## 2. Strict Kinetic Model

### 2.1. Force Analysis

BNNSs in ultrapure water under nanosecond fields are mainly subject to the electric field force.

**Figure 2:**
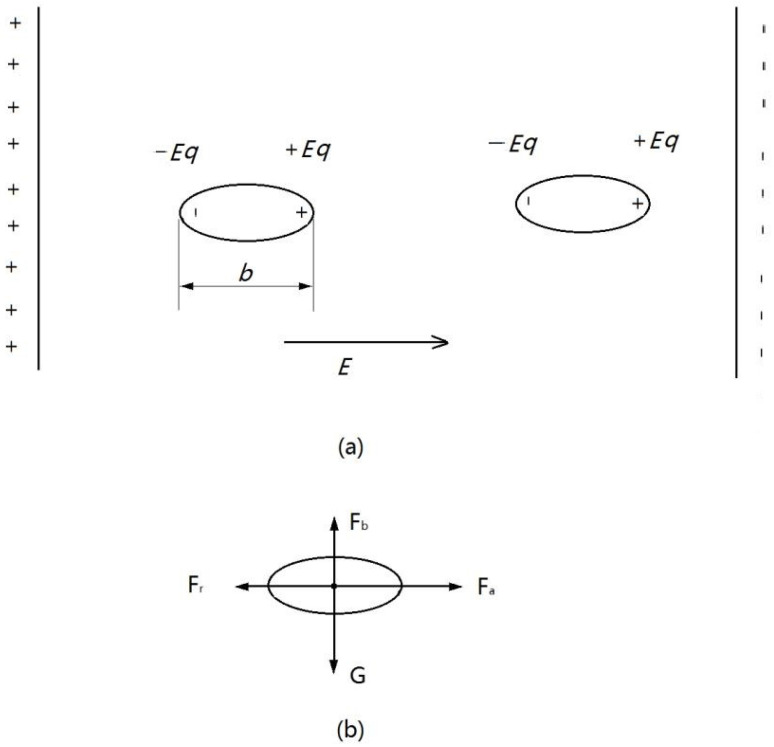
Force analysis for BNNSs under electric field immersed in ultrapure deionized water. (**a**) Global electric force; (**b**) forces imposed on a single BNNS.

First, the global electric field force is analyzed. The configuration of a single BNNS is oval. The two ends along the long axis of the BNNS would have a positive *q* and a negative *q* due to polarization. Therefore, each end of the BNNS in the field of *E* would suffer an electric field force of *Eq*. The two electric field forces tend to draw the BNNS to let its long axis be parallel to the direction of *E*. After that, the sum of the two electric field forces is 0, as shown in Figure 2a.

Then, the forces imposed on a single BNNS are analyzed. Assume that different BNNSs have already stood in a line but are not connected to each other. Generally, a single BNNS would suffer four kinds of forces when a nanosecond pulse is launched: (1) gravity, *G*; (2) buoyancy from the water, *F_b_*; (3) attraction force, *F_a_*; (4) resistance, *F_r_*. These four types of forces are shown in Figure 2b. *G* and *F_b_* together determine the vertical motion process of a BNNS. *F_a_* and *F_r_* together determine the horizontal motion process of the BNNS. Since the sum of *G* and *F_b_* is small compared with the sum of *F_a_* and *F_r_*, only the horizontal motion process of the BNNS is taken into account in this paper.

### 2.2. Strict Model

**Figure 3:**
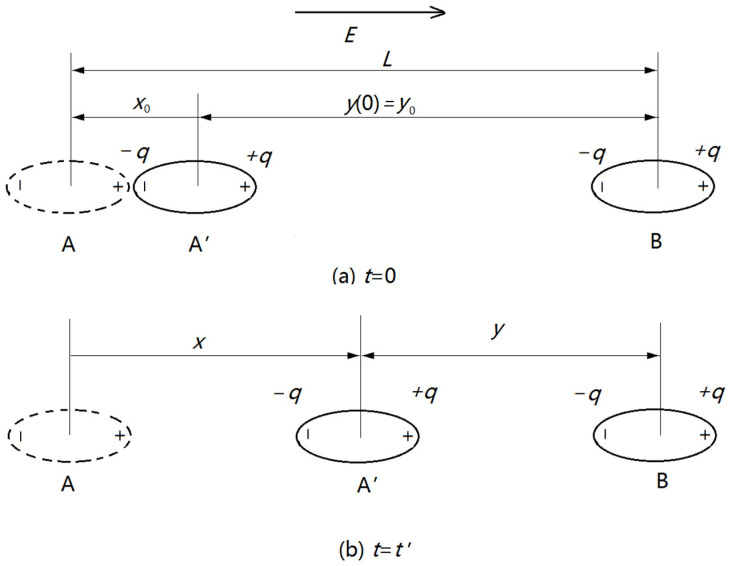
Kinetic model of a BNNS. (**a**) Initial state when *t* = 0; (**b**) mediate state when *t* = *t*′.

To establish a physical kinetics model of a BNNS, the attraction force of two BNNSs is analyzed. Assume the long axis of an oval BNNS is *b* and the center-to-center distance of two BNNSs is *y*; then, as to the left BNNS, A, in Figure 3, it suffers four Coulomb forces, two of them belong to the attraction force, i.e.,:

(2) $$F_{A-B+}=k\frac{q^{2}}{{\left(y+b\right)}^{2}}$$

and

(3) $$F_{A+B-}=k\frac{q^{2}}{{\left(y-b\right)}^{2}}$$

where *k* is the Coulomb constant.

(4) $$k=\frac{1}{4\pi \varepsilon_{0}\varepsilon_{r}}$$

in which *ε*_0_ is the dielectric constant in vacuum; *ε*_r_ is the relative dielectric constant of water. Another two of the Coulomb forces belong to the repulsion force, i.e.,:

(5) $$F_{A-B-}=F_{A+B+}=-k\frac{q^{2}}{y^{2}}$$

where “−” represents the repulsion force. Then, *F_a_* is the sum of the four Coulomb forces expressed in Equations (2)–(5), i.e.,:

(6) $$\begin{array}{l} F_{a}=F_{A+B-}+F_{A-B+}-F_{A-B-}-F_{A+B+}\\ =k\frac{q^{2}}{y^{2}}\left[\frac{1}{{\left(1+b/y\right)}^{2}}+\frac{1}{{\left(1-b/y\right)}^{2}}-2\right]\approx 6kq^{2}b^{2}\frac{1}{y^{4}} \end{array}$$

As to *F_r_*, if the velocity (*v*) of the BNNS is not high enough, *F_r_* is proportional to *v*, i.e.,:

(7) $$F_{r}=-k_{l}v$$

where *k_l_* is a constant which is determined by the object configuration, fluid types, etc.

Assume that BNNS B is static and a one-dimensional coordinate system can be established as follows: the direction from BNNS A to BNNS B is the positive direction; the original position of BNNS A is the zero point; the displacement of BNNS A is *x*. So, *x* + *y* = *L* where *L* is original center-to-center distance between BNNS A and BNNS B, as shown in Figure 3b. Then, the horizontal kinetic motion function is as follows:

(8) $$ma=F_{a}-F_{r}$$

where *a* and *m* are the acceleration and mass of BNNS A, respectively. Taking into account that

(9) $$\begin{array}{cc} a=\frac{d^{2}x}{dt^{2}}, & v=\frac{dx}{dt} \end{array}$$

Equation (8) can be changed to as follows:

(10) $$m\frac{d^{2}x}{dt^{2}}=6kq^{2}b^{2}\frac{1}{y^{4}}-k_{l}\frac{dx}{dt}$$

or

(11) $$\frac{d^{2}x}{dt^{2}}+M\frac{dx}{dt}=N\frac{1}{y^{4}}$$

where *M* = *k_l_*/*m* and *N* = 6 *kq*^2^*b*^2^/*m*. Taking into account that *x* = *L − y*, d*x*/d*t* = − d*y*/d*t* and d^2^*x*/d*t*^2^ = − d^2^*y*/d*t*^2^, Equation (11) can be further changed as follows with *y* as the function:

(12) $$\frac{d^{2}y}{dt^{2}}+M\frac{dy}{dt}=-N\frac{1}{y^{4}}$$

The initial conditions of Equation (12) are as follows:

(13) $$\begin{array}{cc} y{|}_{t=0}=y_{0}, & \frac{dy}{dt}{|}_{t=0}=-v_{0} \end{array}$$

Equation (12) together with the conditions in Equation (13) is the strict kinetic model to describe the motion process of a BNNS in water under nanosecond pulses.

**Figure 4:**
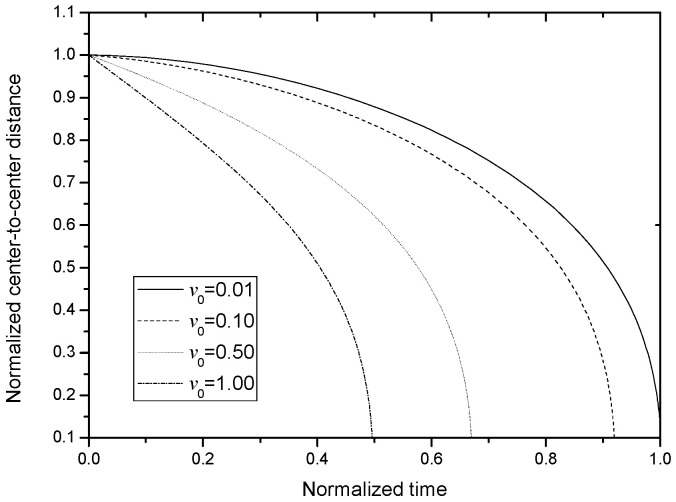
Numerical solution for the strict kinetic model in Equation (12) when *M/N* = 1 and *y*_0_ = 1.

It is noteworthy that no analytical solution to Equation (12) exists. Therefore, a numerical approach was implemented, involving appropriate values of *M*, *N*, *y*_0_, and *v*_0_, as depicted in Figure 4, with the raw program presented in the Appendix A. As shown in Figure 4, the numerical solution curves exhibit a general trend consistent with the experimental data presented in Figure 1. Moreover, the data clearly indicate that as *v*_0_ increases, the time required for *y* to decrease from *y*_0_ to 0.1 reduces progressively. These observations provide further validation of the strict kinetic model employed to describe the motion of BNNSs under nanosecond pulses.

## 3. Simplified Mathematical Model

### 3.1. Simplified Model

Even though the kinetic model in Equation (12) ideally reflects the basic motion process of a BNNS, it produces no analytical solution. In addition, there is no relation between the fitting parameters and the motion characteristics. To overcome these shortcomings, a simplified kinetic model is proposed based on the following fact: the shorter the distance, the faster the BNNS moves due to the attraction force.

In perspective of mathematics [20], that the shorter is the distance between BNNS A and BNNS B means the increase of *x*; that the faster the BNNS A moves means that its *v* is positively correlated to *x*. Here, it is assumed that *v* is simply proportional to *x*, i.e.,:

(14) $$\frac{dx}{dt}=k_{t}x$$

where *k_t_* is a constant. It has the following physical meaning: the larger *k_t_* is, the faster a BNNS moves. Also taking into account that *x* = *L* − *y* and d*x*/d*t* = − d*y*/d*t*, Equation(14) can change to the following, with *y* as the function:

(15) $$\frac{dy}{dt}=-k_{t}\left(L-y\right)$$

The initial condition of Equation (15) is

(16) $$y{|}_{t=0}=y_{0}$$

Equation (15) together with the condition in Equation (16) is the simplified kinetic model to depict the motion process of a BNNS in water under nanosecond pulses.

### 3.2. Analytical Solution

**Figure 5:**
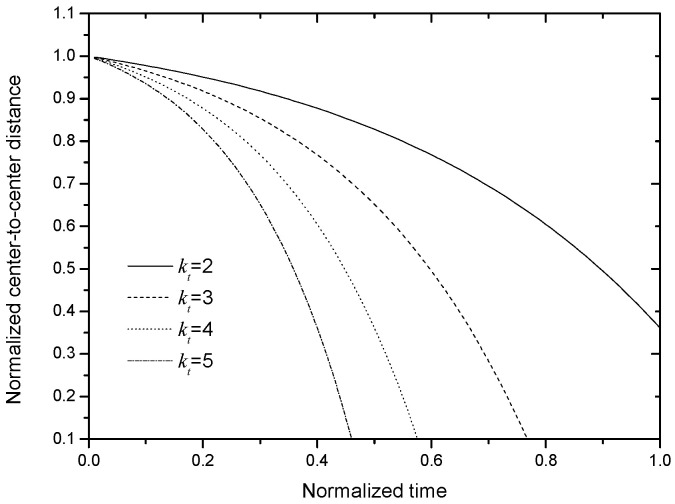
Analytical solution for the simplified kinetic model in Equation (17) when *L* = 1.1 and *y*_0_ = 1.

Equation (15) can produce an analytical solution as follows:

(17) $$y\left(t\right)=L-\left(L-y_{0}\right)\exp\left(k_{t}t\right)$$

or simply

(18) $$y\left(t\right)=L-C\exp\left(k_{t}t\right)$$

where *C* = *L* − *y*_0_. Figure 5 plots the curves of the center-to-center distance, *y*, on *t* for different *k_t_*. From this figure, it is seen that (1) the tendencies of *y* on *t* basically agrees with those shown in Figure 4, so, it can reflect the physical process shown in Figure 1; and (2) a larger *k_t_* corresponds to a shorter alignment time, which agrees with the basic physical fact.

Now, by comparing the analytical solution in Equation (18) with the presumed fitting function of Equation (1), some comments can be made on Equation (18):

Firstly, Equation (18) describes an exponential decrease process. In an exponential decrease process, the exponent in exp(⋅) is positive and the function, *y*, decreases faster and faster as *t* increases. But the exponent in exp(⋅) in Equation (1) is negative, which means that *y* decreases slower and slower as *t* increases.

Secondly, the physical meanings of each parameter in Equation (18) are clear whereas those in Equation (1) are not. For example, *L* is the original center-to-center distance between BNNS A and BNNS B; *y*_0_ ( = *L* − *C*) is the initial center-to-center distance; *k_t_* is the proportional factor between the velocity (*v*) and the displacement (*x*) of BNNS A, and the larger *k_t_* is, the faster *y* decreases.

Lastly, other physical parameters to describe the motion process can be easily deduced based on Equation (18) but cannot be deduced from Equation (1). For example, the total time, Δ*t*, for *y* to decrease from the initial value, *y*_0_, to a fixed value, such as *b* (the long axis of the oval BNNS), can be deduced as follows:

(19) $$\Delta t=\frac{1}{k_{t}}\ln\left(\frac{L-b}{L-y_{0}}\right)$$

In addition, the initial velocity, *v*_0_, and the acceleration, *a*, of the BNNS can also be deduced as follows:

(20) $$v_{0}=\frac{dy}{dt}{|}_{t=0}=-k_{t}\left(L-y_{0}\right)$$

and

(21) $$a=\frac{d^{2}y}{dt^{2}}{|}_{t=0}=-k_{t}^{2}\left(L-y_{0}\right)$$

These are the advantages of the simplified kinetic model.

## 4. Practical Application

**Figure 6:**
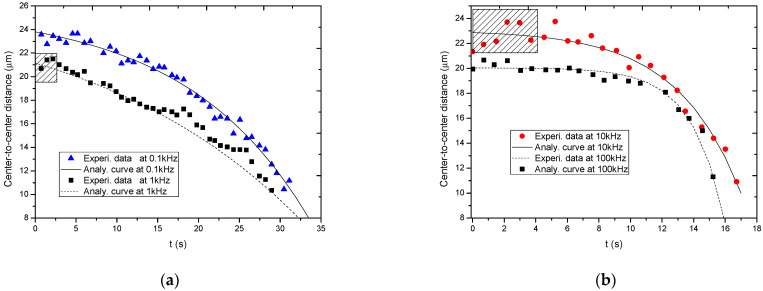
Fitting of the experimental data with the analytical solution. (**a**) Frequency of 0.1 kHz and 1 kHz. (**b**) Frequency of 10 kHz and 100 kHz.

Firstly, Equation (18) is used to re-fit the experimental data in Ref. [4], and the fitting results are shown in Figure 6. From these two Figures, it is seen that the analytical curves fit well the experimental data, which verifies the correctness of the simplified kinetics model for the BNNSs’ motion process in ultrapure deionized water under nanosecond pulses.

**Figure 7:**
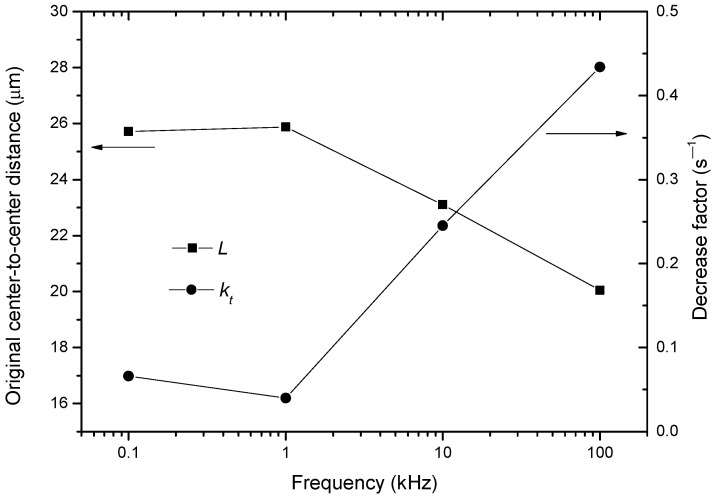
Dependencies of *L* and *k_t_* under different frequencies.

Secondly, the fitting parameters based on Equation (18) under different frequencies are summarized, as listed in Table 1. Based on the data in Table 1, the tendencies of the original center-to-center distance, *L*, and the decrease factor, *k_t_*, on the pulse frequency are plotted, as shown in Figure 7. From this figure, it is seen that *L* ranges from 20 to 25 μm; and *y*_0_ ranges from 20 to 23 μm. In addition, the decrease factor, *k_t_*, tends to increase as the frequency increases. This agrees with the fact that the BNNS becomes more agile at a higher frequency.

**Figure 8:**
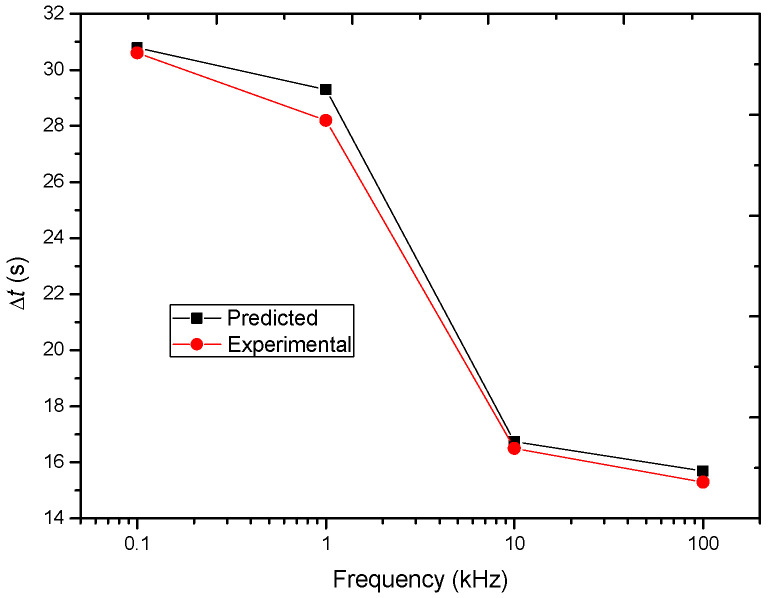
Comparison between the predicted time consumed for motion and the experimental time under different frequencies.

**Figure 9:**
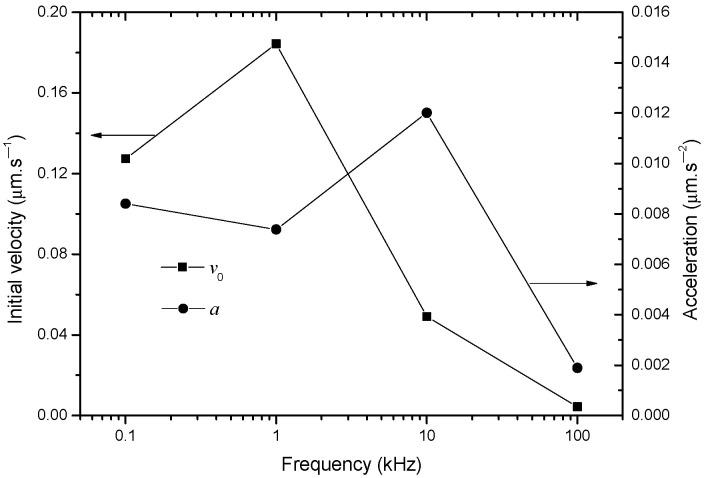
The initial velocity and acceleration of BNNS under different frequencies.

Lastly, the total motion time, Δ*t*, can be predicted based on Table 1 and Equation (19), which are listed Table 2. The final center-to-center distance, *b*, of BNNSs is set as 11 μm, which is the average long axis of the oval BNNS, as shown Figure 3b. The experimental Δ*t* for *y* to decrease from *y*_0_ to *b* is also measured and listed in Table 2. Figure 8 compares these two types of values. From this figure, it is seen that the predicted values agree well with the experimental values, which further support the correctness of the simplified model. In addition, Table 2 also lists the value of |*v*_0_| and |*a*| of the BNNSs in the initial motion process based on Equations (20) and (21), respectively. Figure 9 plots the tendencies of these two parameters on frequency. From this figure, it is seen that as the pulse frequency increases, |*v*_0_| tends to decreases but |*a*| is in a range of (2–12) × 10^−3^ μm × s^−2^.

## 5. Remarks

### 5.1. Relation between Initial Center-to-Center Distance y_0_ and Original Center-to-Center Distance L

A simplified kinetic modelkinetic model to describe the motion process of BNNSs under nanosecond pulses is presented, which not only satisfactorily fitted the experimental data but also gave more information about the motion process. However, there is still a question. In Ref. [4], it was observed that the center-to-center distance of the two BNNS particles sometimes slightly increases in the beginning and then gradually decreases until aligning in a line, indicating that the two particles first repel each other and then attract each other. This is because once the particles repel each other, the center-to-center distance increases slightly. This phenomenon is not only obviously shown in the shadow regions in Figure 5a for the frequency of 1 kHz but also shown in the shadow region in Figure 5b for the frequency of 10 kHz.

From the perspective of the simplified mathematical model, this phenomenon can be explained more clearly. Based on the data in Table 1, it is found that the initial center-to-center distance of *y*_0_ is not equal to the original center-to-center distance of *L* at all. This is correct, since *y*_0_ must not be equal to *L* or else Equation (15) would degrade to

(22) $$\frac{dy}{dt}=0$$

**Figure 10:**
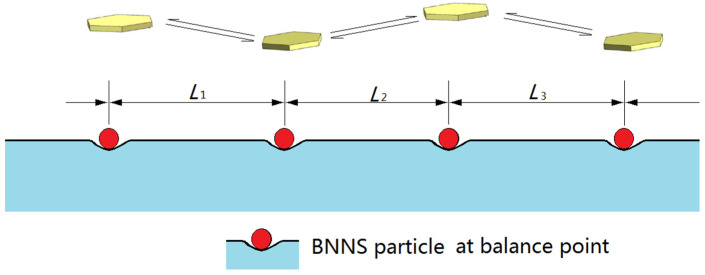
Schematics of stable points for the BNNSs when immersed in water.

The solution for Equation (22) is that *y*(*t*) = *y*_0_ = *L*, which means that the particle of a single BNNS will be static and never move. In other words, *y* = *L* is the stable point or balance position of the system. In order to connect in a line, all the BNNS sheets must overcome these stable points to get attracted or repelled each other, as shown in Figure 10.

The stable points of the system are ‘fragile’ and can be easily destroyed. When a series of repetitive nanosecond pulses are imposed on these BNNSs, the BNNSs suffer perturbation. Some of them get far away from each other; some of them directly attract each other. In other words, the metastable states are destroyed in the initial stage and the particles begin to accelerate to move toward each other until aligning in a line.

### 5.2. Effect of Pulse Duration on BNNSs’ Motion Behavior

It is mentioned in Section 1 that the advantage of using nanosecond pulses to research the properties of BNNSs lies in the weak heat effect and the discrete electric field duration. Obviously, the shorter the pulse duration, the weaker the heat effect and the more advantageous this method is. However, there is a lower limit of the pulse duration since the start of the motion of a BNNS needs a time, *t*_0_. Only when the pulse duration, *τ*, is longer than *t*_0_, can the motion of the BNNS be triggered; otherwise, the BNNS would be motionless. By far, the minimum value of *t*_0_ obtained by Y. Mi et al. is tens of nanoseconds. Whether a duration as short as one on a picosecond and femtosecond scale can trigger the motion of a BNNS should be explored in future studies.

## 6. Conclusions

The motion process of BNNSs immersed in ultrapure deionized water under nanosecond pulses is analyzed. A strict kinetic model is established, which is a linear second-order ordinary differential equation but has no analytical solution. As a substitution, a simplified mathematical model is established based on the fact that the shorter the center-to-center distance, the faster the BNNS moves. With this simplified model, the center-to-center distance of BNNSs dependent on time is presented in an analytical way, which fits well the experimental data. In addition, the total motion time, the velocity, and acceleration of BNNSs in the motion process are all deduced. The simplified kinetic model can better describe and explain the motion behavior of the BNNSs in water under nanosecond pulses and can give more information about the motion.

Because the simplified model is used to describe the residual distance between two BNNSs, it is named the ‘Residual Model’. This model can also be used to predict the residual population when a pest invades into a region.

## Figures and Tables

**Table 1:** Fitting values for the BNNS under nanosecond pulses.

Frequency, kHz	*L*, μm	*C*, μm	*y*_0_ = *L* − *C*, μm	*k_t_*, s^−1^
0.1	25.72	1.94	23.78	0.066
1	25.88	4.62	21.26	0.040
10	23.10	0.02	23.08	0.245
100	20.05	0.01	20.04	0.434

**Table 2:** Deduced values for the BNNSs under nanosecond pulses.

Frequency, kHz	Δ*t*, s (*b* = 11 μm)		|*v*_0_|, μm × s^−1^	*|a*| × 10^−3^, μm × s^−2^
	Predicted Value	Experimental Value		
0.1	31.8	31.5	0.13	8.4
1	30.9	29.1	0.18	7.4
10	17.1	16.7	0.05	12
100	15.9	15.5	0.004	1.9

## Data Availability

Data presented in this study are available on request from the second author.

## Appendix A

This attachment gives the program in Matlab to solve Equation (12) together with the conditions in Equation (13), which includes the definition of Equation (12) and the main program.

### Appendix A.1. Definition of Equation

function dy = F(t,y)
M = 1;
N = 1;
dy = [y(2); − N × y(1).^(−4) – M × y(2)];
end

### Appendix A.2. Main Program

y0 = 1;
v0 = 0.1;
[T, Y] = ode45(@F,[0,1.5],[y0;−v0]);
plot(T,Y(:,1),’−+’)
hold on
